# Molecular Fractionation of Ancient Organic Compounds in Deeply Buried Halite Crystals

**DOI:** 10.1021/acs.analchem.4c02956

**Published:** 2024-10-10

**Authors:** Xiuyan Liu, Odile Barres, Jacques Pironon, Miriam Unger, Pierre Beck, Junjia Fan, Mehdi Ostadhassan

**Affiliations:** † Institute of Energy, Peking University, Beijing 100871, China; ‡ Université de Lorraine, CNRS, GeoRessources Lab, F-54506 Vandœuvre-lès-Nancy, France; § Photothermal Spectroscopy Corporation, Santa Barbara, California 93101, United States; ◇ Univ. Grenoble Alpes, CNRS, IPAG, 38000 Grenoble, France; ◧ Research Institute of Petroleum Exploration and Development, RIPED, Key Laboratory of Basin Structure and Hydrocarbon Accumulation, China National Petroleum Corporation, Beijing 100083, China; □ Institute of Geosciences, Christian-Albrechts-Universität, Kiel 24118, Germany

## Abstract

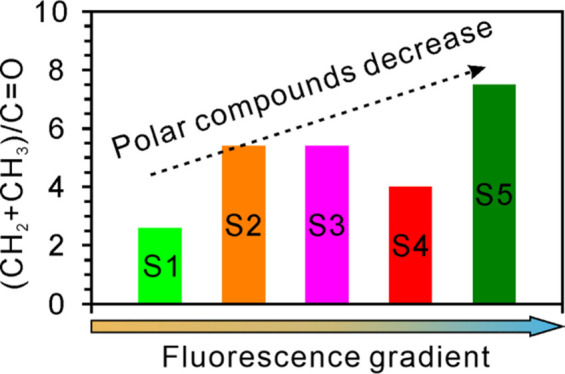

The molecular fractionation of organic compounds through adsorption in minerals has wide implications, including tracing the origins of life, carbon sequestration, and climate change. Here we present the first in situ examination of molecular fractionation within individual crystals via optical-photothermal infrared (O-PTIR) spectroscopy. Our study focuses on a unique inclusion trail within deeply buried halite crystals, characterized by a distinctive orange-to-blue fluorescence gradient, providing primary evidence of molecular variation in ancient carbon-based fluids within the inclusion trail. The findings reveal a trend in the CH_2_/C=O and CH_3_/C=O ratios, conforming with a consistent decrease from the blue fluorescence region to the orange fluorescence region. The chemically influenced fluorescent behavior of these ancient liquid carbon-based compounds is attributed to the fractionation of fluids in the inclusions as a result of microfractures within the crystal acting as chromatography capillaries. These capillaries facilitated interactions between specific organic compounds, serving as adsorbates, and the halite mineral, representing the adsorbent. Our study provides insights into the fluid–solid physicochemical interactions within extreme environments and extends our understanding of molecular processes in such settings.

## Introduction

Carbon-based fluid inclusions in natural minerals open the door to the ancient fluid history of the sedimentary environment and have been widely used in geological sciences, such as to trace the origin of life on earth,^1^ possible life analogues on Mars,^2^ movement of subsurface carbon based fluids,^3^ CO_2_ sequestration,^4^ and records of climate change.^5^ It has been long considered that exposure of ancient carbon-based fluids to higher pressure and temperature in the subsurface would alter the composition which has been hypothesized to manifest itself in fluorescence spectral parameters.^6−9^ However, since other factors like UV irradiation,^10^ water washing,^8^ trapping fractionation,^8^ degradation,^7^ and migration fractionation^11^ can affect fluorescence colors, evaluating organic fluids’ thermal history based solely on fluorescence color remains questionable. Molecular fractionation of ancient fluid organic compounds has been investigated through conventional methods^12^ but has not been directly observed. Recently, a trail of oil inclusions (<20 μm) detected in deeply buried ancient halite crystals that dated back to the Eocene, with fluorescence colors gradually changing from orange to blue,^13^ provides an ideal opportunity for investigating factors controlling the fluorescence color of inclusions and provides direct evidence of organic compound fractionation in a single mineral. Confirming this would require chemical information on the inclusion via spectroscopy or chromatography methods which has not been possible to date for extremely small targets without altering the assemblages, either via Fourier transform infrared (FTIR) spectroscopy,^3^ chromatography or comparisons of chemical compounds^14,15^ between two separated fractions (inclusions). The size of the inclusion and diffraction limit in analytical methods have been the major obstacles to obtain the direct indication to relate fluorescence color to the inclusion chemical composition in situ.^16^

Recent advances in IR spectroscopy have overcome the Abbe diffraction limit and achieved a nanoscale resolution (∼20 nm), via atomic force microscopy-based-infrared spectroscopy (AFM-IR)^17^ with successful applications in geosciences, including resolving carbonaceous inclusions armored within garnet porphyroblasts from metasedimentary rocks^1^ or delineating nanoscale chemomechanical properties of organic matter in clay-rich fine-grained sedimentary rocks.^18,19^ However, AFM-IR spectroscopy requires a certain flatness of the sample surface and the targeted substance should be close to the surface (<1 μm),^17^ and both can hinder the analysis of inclusions. Optical-photothermal infrared spectroscopy (O-PTIR) is a newly developed noncontact mode of IR technique,^20^ which minimizes sample preparation, avoids cross-contamination, and is capable of mapping the surface composition of organic matter at the nanometer scale.^21^

In this study, we present the first application of O-PTIR in analyzing inclusions hosting carbon-bearing fluids (trapped at around 6.6 Ma) to provide robust, in situ chemical information at nanoscale, as a result of molecular fractionation within a single mineral. The O-PTIR maps are correlated with detailed fluorescence and transmission microscopy images to locate the fluid inclusions during the O-PTIR measurements. This technique allows resolving the chemical composition of the inclusions (<20 μm) with varying fluorescence colors in the same assemblage and reveals the chemical evolution with varying fluorescence color along the trapping trail, proving that variations in the fluorescence color are the result of molecular fractionation due to the interactions between the organic compounds as the adsorbate and halite crystal representing the adsorbent.

## Materials and Methods

The oil inclusion trail examined here occurs in a halite crystal aliquot (<2 mm thick, <6 mm across) that was separated from a larger salt rock core sample that was retrieved from around 3,285 m depth in the third member of the Shahejie Formation (Eocene) in the northern Dongpu Depression, Bohai Bay Basin, China.^13^ The halite chip was then mapped by optical microscopy for locating each fluid inclusion in the trail on the *X*–*Y* plane for correlative microscopy. Then, the O-PTIR spectra and molecular distribution maps were collected by using a mIRage infrared microscope. The visible laser power was 6.5%, and the IR laser power was 78%. IR point spectra were measured on and outside the inclusion trail based on optical microscopy observations.

## Results

### Optical Microscopy

**Figure 1:**
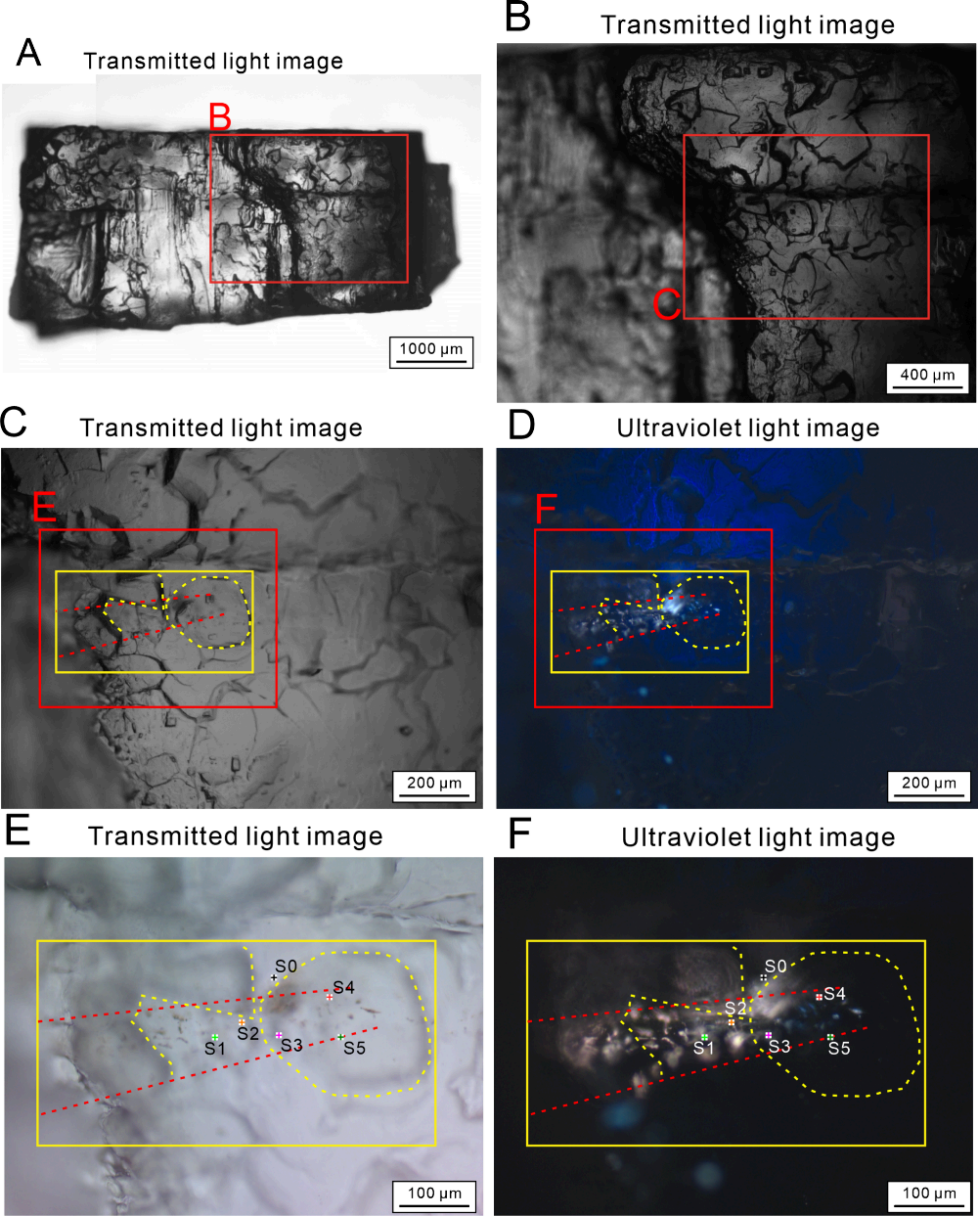
Optical images showing the location of the oil inclusion trail in the halite crystal. (A) White transmitted light image (2× objective) of the halite crystal. (B) White transmitted light image (5× objective) of the ROI. (C) White transmitted light image of the same field of view as that of panel D. (D) UV light image of fluorescing inclusions shifting from blue (right) to orange (left) along the trail. (E) White transmitted light image of irregular inclusion shapes along the trail. (F) UV light image of fluorescing inclusions varying from blue (right) to orange (left) along the trail, the same field view as that of panel E.

Optical observations under white transmitted light and incident UV light revealed the presence of organic compounds within the inclusion trail in the halite crystal (Figure 1A–F). The halite crystal sample is around 6 mm in length and 3 mm in width (Figure 1A). The ROI is located on the protruding part of the sample surface (Figure 1B). The fluorescence colors of the fluid inclusions show a gradual change from blue (right) to orange (left) along the trail, which was used as the primary evidence of molecular variations (Figure 1C–F) that are cross-cut by the boundary of the protruding part of the sample surface (Figure 1C–F). More detailed description about the sample can be found in Liu et al.^13^

### O-PTIR Chemical Mapping

**Figure 2:**
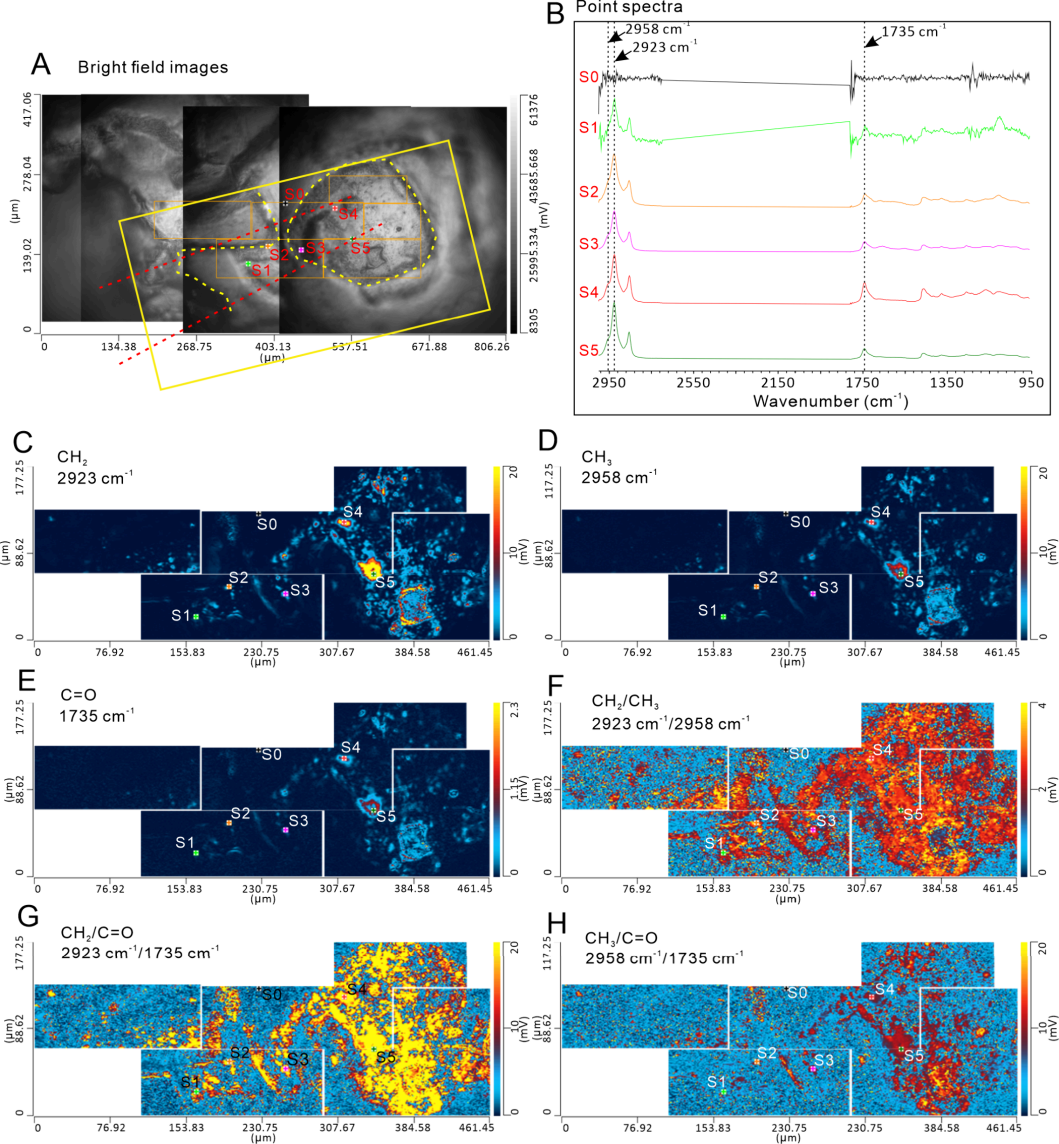
O-PTIR images of the ROI. (A) Bright field images showing the location of the ROI. (B) Point spectra for the selected points marked in red, pink, orange, light green, dark green, and black on the different images. (C) Chemical maps of CH_2_ concentration. (D) Chemical maps of CH_3_ concentration. (E) Chemical maps of C=O concentration. (F) Maps of the CH_2_/CH_3_ ratio. (G) Maps of CH_2_/C=O ratio. (H) Maps of CH_3_/C=O ratio.

The bright field images (Figure 2A) were acquired before IR acquisition for comparison with the optical images (Figure 1) to locate the ROI. Six O-PTIR point spectra were collected at selected locations in the ROI (Figure 2B). They correspond to the points marked in red, pink, orange, light green, dark green, and black on the different images (Figure 2A–H). The black spectrum (S0) is recorded outside the inclusion trail in the halite crystal. It shows no IR absorption modes since halite is transparent in the mid-IR. The five other IR spectra recorded in the fluid inclusion trail show the same combination of absorption bands. They feature a well-defined combination of bands between 3000 and 2800 cm^–1^ related to stretching from the methyl CH_3_ and methylene CH_2_ groups. The other bands correspond to the carbonyl C=O groups at 1735 cm^–1^, to the methylene bending vibration in C–CH_2_ at 1462 cm^–1^, to the OH bending vibration at 1410 cm^–1^, to the methyl bending vibration in C–CH_3_ at 1377 cm^–1^, and to the stretching vibrations C–O in =C–O–C at 1260 cm^–1^ and in C–O–C at 1170 and 1107 cm^–1^. The pink spectrum (S3) shows two other bands at 1655 and 1551 cm^–1^ corresponding respectively to the C=O stretching vibration and to the N–H bending vibration in secondary amides.^22^ The relative intensities of the bands present in all of the spectra are not the same, indicating differences in the composition of the organic matter. Area calculations of the bands corresponding to the CH_2_ and CH_3_ groups (baseline 3000–2800 cm^–1^) and to the C=O group (baseline 1800–1696 cm^–1^) were carried out on the five point spectra. The area ratios (CH_2_+CH_3_)/C=O are 2.6, 5.4, 5.4, 4.0, and 7.5 for the light green (S1), orange (S2), pink (S3), red (S4), and dark green (S5) points.

Six rectangular areas in the ROI were scanned by an O-PTIR spectrometer by tuning the IR laser at 2958 cm^–1^ for CH_3_ asymmetric stretching maps, at 2923 cm^–1^ for CH_2_ asymmetric stretching maps, and at 1735 cm^–1^ for C=O carbonyl group stretching maps (Figure 2). The CH_2_ maps (Figure 2C) show some high energy signals in the right part of the ROI, mostly with cyan color, and fading toward the bottom left. CH_3_ maps (Figure 2D) and C=O maps (Figure 2E) generally exhibit patterns similar to those of CH_2_ maps with lower energy. These images clearly demonstrate the distribution of different functional groups in the areas in the ROI at a very good spatial resolution. CH_2_/CH_3_ maps present generally strong IR signals in the right part, and the signals decrease to background level toward to the bottom left (Figure 2F). CH_2_/C=O (Figure 2G) and CH_3_/C=O (Figure 2H) maps exhibit the same trend with a higher energy for CH_2_/C=O due to the higher concentration of CH_2_ groups. Panels G and H of Figure 2 make it possible to visualize the variations of relative intensities of the band areas and indicate an increase in the concentration of the CH_2_ and CH_3_ groups compared with the C=O groups.

## Discussion

The inclusions measured in this study occur as a trail (or healed fracture plane) as deduced from petrographic observation, and therefore, they are considered to belong to a single fluid inclusion assemblage (FIA).^23^ The fluorescence color variation indicates that the chemical compositions (ratio among saturates, aromatics, resins, and asphaltenes) of these organic compounds are different,^24^ suggesting the FIA has either undergone fractionation during trapping^8^ or post-trapping alterations (reequilibration).^25^ The UV irradiation has been proved to have significant influence on the fluorescence color of carbon-based fluid inclusions, and often a red shift occurs, particularly in KCl crystals^10^ and dolomite.^26^ This effect can occur once the UV exposure begins and induces significant irreversible changes within a few minutes.^10,26^ However, the UV light observation is necessary for the identification of organic fluids within inclusions^27^ and was therefore not eliminated in this study. Thus, it was considered that the fluorescence colors of the fluid inclusions have already been altered before the O-PTIR measurements. In comparison with the images in our previous work,^13^ the saturation of fluorescence color of the inclusions decreased; however, the gradient from a longer wavelength to a shorter wavelength along the inclusion trail, which is the target phenomenon in this study, is still observed (Figure 1). Therefore, it is considered that although the UV alterations on the inclusion trail have occurred, this has not significantly eliminated the fluorescence color gradient along the trail because the irradiation time has not been long enough to cause the entire organic compounds within the minerals to fluoresce the same color.^26^

Water washing,^8^ gas washing,^28^ and pressure release^29^ derived phase separation of organic compounds have been reported to cause alterations in the fluorescence color. These phenomena are more likely to occur in the original habitat where the organic fluids are exposed to pressure and temperature in reservoirs rather than in the inclusions after trapping. Moreover, the inclusions in single FIA that formed after phase separation where the fluids have been accumulated in the pore spaces generally exhibit a similar fluorescence property.^28,29^ Though FIAs containing variable fluorescence colors have also been found in such conditions as a result of washing, no petrographically preferential distribution of the fluorescence has been reported.^28^ Therefore, the fluorescence gradient along the FIA in this study is not likely to have resulted through the phase separation of organic compounds in their original form.

Biodegradation will preferentially remove lower molecular *n*-alkanes in liquid hydrocarbons, and the resultant decrease in the aliphatic/aromatic fraction ratio and increase in the polar fractions will induce a red shift of fluorescence color.^8^ Organic compounds within inclusions with orange fluorescence color has been suspected to undergo biodegradation before trapping.^9^ However, previous studies suggest that biodegradation may not possibly occur in such fluids in the Shahejie Formation strata because the formation temperature and burial depth exceeded favorable biodegradation zone when the conversion of solid organic material to liquids began.^30^

**Figure 3:**
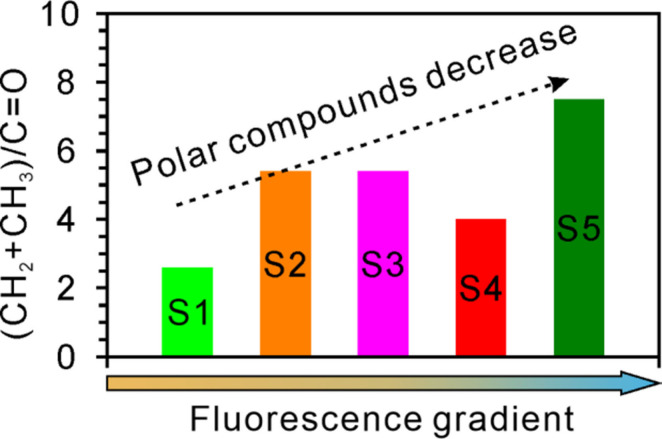
(CH_2_+CH_3_)/C=O ratios of five-point spectra along the fluorescence gradient from orange (left) to right (blue).

Trapping fractionation is a commonly mentioned phenomenon to account for fluorescence color variation, but no detectable difference has been found in chemical compositions of ancient carbon-based fluid inclusions from a single source.^6,8,10,27,31^ This is because polar fractions in hydrocarbons are more preferentially adsorbed onto minerals’ surface than aromatics and saturates during charging, which is also the main principle of geochromotography or molecular fractionation.^32^ In sandstone layers, fractionation as a result of geochromotography is usually observed across the length of meters to kilometers,^33−35^ but in clay-organic-liquid-rich fine grained sediments, fractionation due to the mobility of organic compounds can occur during the process of conversion of organic material to other fluids.^31,32^ In this study, the FIA shows a fluorescence color gradient from orange to blue (Figure 1), with the increase in CH_2_ and CH_3_ concentrations to C=O that are revealed by O-PTIR (Figure 2) along the fluorescence color gradient. It is inferred that the preferential adsorption of polar and aromatic fractions on the mineral surface (fracture wall in halite) controls the fluorescence color changing. The (CH_2_+CH_3_)/C=O ratios calculated from the five point spectra also show a rough increase from orange fluorescence to blue fluorescence (Figure 3). The energy response of CH_2_/C=O, CH_3_/C=O, and CH_2_/CH_3_ that are clearly delineated in the right part of the ROI does not align with the distribution pattern of the liquid inclusions. This is interpreted as organic films that are undetectable using conventional fluorescence microscopy but that are visible by the O-PTIR technique. These microfractures, as well as the major fracture that is responsible for the formation of such ancient inclusion trail, were acting as chromatography capillaries. When a small amount of these hydrocarbons charge into these fractures, fractionation occurs and chemical changes along the charging direction are produced. After fracture healing following the trapping of the organic compounds as inclusions, their varying chemical compositions are revealed by a fluorescence color gradient along the trail as the primary evidence.

Quantitative relationships between fluorescence spectral parameters and variations in the chemistry of organic compounds trapped within a single mineral have been constructed from fluids that have not experienced biodegradation, water washing, phase separation, and trapping fractionation.^9^ In this study, the inclusion trail (one FIA) represents an instant trapping event of organic fluids that are expelled from the underlying layers that were rich in organic material after exposure to heat due to increase in the burial depth, with their temperature well constrained within tens of degrees.^36^ Thus, it is impossible for several stages of conversion of organic matter to liquids to have occurred to cause the entrapment of the fluids of various chemical composition simultaneously.

## Conclusion

Our study reports the first application of the O-PTIR technique to ancient inclusions rich in organic compounds in situ without altering the assemblage. We present direct evidence of variations in the fluorescence color of carbon-based liquids in a single crystal of halite, dated back to the Eocene, related to changes of chemical compositions of the content. Our study recommends further investigations on physicochemical interactions between minerals and organic material in harsh environments to understand adsorption phenomenon (liquid chromatography events in subsurface) and also demonstrates the advantages of using submicrometer IR techniques in inclusion analysis with application to climate change and energy transition.

## Supporting Information Available

Sample preparation, optical microscopy, and optical-photothermal infrared spectroscopy (O-PTIR) (PDF)
