# Supplementary material for: Molecular Fractionation of Ancient Organic Compounds in Deeply Buried Halite Crystals
Source: Anal Chem. 2024 Oct 10;96(42):16493–8. doi: 10.1021/acs.analchem.4c02956 (PMC11503512; doi:10.1021/acs.analchem.4c02956)
Supplement: Supplementary file 1 — ac4c02956_si_001.pdf [file ac4c02956_si_001.pdf]

Supplementary Material of

Molecular fractionation of ancient organic compounds in deeply buried halite crystals

Xiuyan Liu[1], Odile Barres[2], Jacques Pironon[2], Miriam Unger[3], Pierre Beck[4], Junjia Fan[5], Mehdi Ostadhassan[6*]

[1]*Institute of Energy, Peking University, Beijing 100871, China*

[2]*Université de Lorraine, CNRS, GeoRessources Lab, F-54506 Vandœuvre-lès-Nancy, France*

[3]*Photothermal Spectroscopy Corporation, Santa Barbara, CA 93101, USA*

[4]*Univ. Grenoble Alpes, CNRS, IPAG, 38000 Grenoble, France*

[5]*Research Institute of Petroleum Exploration and Development, RIPED, Key Laboratory of Basin Structure and Hydrocarbon Accumulation, China National Petroleum Corporation, Beijing 100083, China*

[6]*Institute of Geosciences, Christian-Albrechts-Universität, Kiel 24118, Germany*

[*]Corresponding author: M. Ostadhassan (mehdi.ostadhassan@ifg.uni-kiel.de)

The supplementary material includes 1) sample preparation, 2) optical microscopy, and 3) optical-photothermal infrared spectroscopy (O-PTIR).

Sample Preparation

A large core sample from the salt caprock (~20 cm in length and ~10 cm in diameter) was retrieved from the P63 Well at a depth of around 3285 m from the Es_3. The location of the well is marked with a red dot in Figure 1C. The core is pure halite with light yellow to white in color, and the size of the halite crystals are generally less than 1 cm. A hammer (10 cm long, 100 g weight) was used to crush the core sample into smaller pieces from the inner parts to avoid surface pieces with potential alteration caused by drilling and associated fluids. These pieces were further cut into smaller chips (< 2 mm thick, < 5 mm across) by a palette knife along the cleavage planes [1, 2].

Optical Microscopy

The halite chip was put on a glass section to observe and locate the oil inclusion trail under transmitted light and incident ultraviolet light using a Zeiss©AX10 microscope equipped with an HBO-100 epi-fluorescence source filtered at 365 ± 5 nm and an LP400 nm emission filter at GeoRessources Laboratory (Nancy, France). The whole sample was mapped under a magnification of 20×. The region of interest (ROI) was subsequently mapped under a range of magnifications (50×, 100×, 200×, 500× and 1000×). The maps were used for locating each oil inclusion in the trail on the X-Y plane for correlative microscopy. The depth of the inclusions to the sample surface was estimated by adjusting the focal plane from the sample surface to the inclusion.

Optical-Photothermal Infrared Spectroscopy (O-PTIR)

The O-PTIR spectra and molecular distribution maps were collected using a mIRage infrared microscope at the Photothermal Spectroscopy Corporation in Santa Barbara, California. Absorption by a sample of infrared photons with a fixed frequency visible wavelength laser induces photothermal effects measured by O-PTIR [3]. In this case, the

spatial resolution limit is set by the wavelength of the visible laser at 532 nm. The ROI was identified in the transmitted light mode with a 10× objective according to the fluorescence and transmission maps obtained by optical microscopy. Following this, the inclusion trail was checked again by using a range of filter cubes for fluorescence integrated in the system under a 40× objective, including DAPI (excitation at 390 ± 9 nm, emission at 460 ± 30 nm), AF488 (excitation at 482 ± 9 nm, emission at 520 ± 14 nm), MCHE (excitation at 578 ± 10.5 nm, emission at 641 ± 37.5 nm), XPol (excitation at 497 ± 8 nm, emission at 535 ± 11 nm), AF647 (excitation at 628 ± 20 nm, emission at 692 ± 20 nm), GFP (excitation at 469 ± 17 nm, emission at 525 ± 19.5 nm) and AutoFluor (excitation at 365 ± 10 nm, emission at 440 ± 300 nm). Subsequently, the molecular distribution maps were measured without baseline deduction. The visible laser power was 6.5% and the IR laser power was 78%. Measurements were conducted in the reflection mode to analyze the photothermal effects of the visible laser, resulting in the acquisition of FTIR transmission spectra. These spectra provide an indirect measurement of the absorbed IR irradiation. The data was acquired as analyzed in the PTIR Studio software. Maps were collected using infrared wavenumbers of 2923 cm^{-1} (CH_2 methylene asymmetric stretching), 2958 cm^{-1} (CH_3 methyl asymmetric stretching) and 1735 cm^{-1} (C=O carbonyl stretching). Branching ratio (CH_2/CH_3) maps were calculated by dividing the CH_2 maps by the CH_3 maps. Individual infrared spectra have a spectral resolution of ≈ 4 cm^{-1}. Point spectrum S0 (black) was obtained outside of the fluorescence area and it shows no chemical responses in CH_2, CH_3 and C=O stretching ranges, indicating the sample contains no CH_2, CH_3 or C=O components except in the oil inclusions. Therefore, the energy intensity on the O-PTIR maps is only caused by CH_2, CH_3 or C=O components in the fluid inclusions.

References Cited

(1) Zhang, H.; Liu, C.; Zhao, Y.; Mischke, S.; Fang, X.; Ding, T. Quantitative temperature records of mid Cretaceous hothouse: Evidence from halite fluid inclusions. *Palaeogeography, Palaeoclimatology, Palaeoecology* **2015**, *437*, 33-41.
(2) Roberts, S. M.; Spencer, R. J. Paleotemperatures preserved in fluid inclusions in halite. *Geochimica et Cosmochimica Acta* **1995**, *59* (19), 3929-3942.
(3) Zhang, D.; Li, C.; Zhang, C.; Slipchenko, M. N.; Eakins, G.; Cheng, J.-X. Depth-resolved mid-infrared photothermal imaging of living cells and organisms with submicrometer spatial resolution. *Science advances* **2016**, *2* (9), e1600521.
